# Inactivation of MARCH5 Prevents Mitochondrial Fragmentation and Interferes with Cell Death in a Neuronal Cell Model

**DOI:** 10.1371/journal.pone.0052637

**Published:** 2012-12-19

**Authors:** Lei Fang, Charles Hemion, David Goldblum, Peter Meyer, Selim Orgül, Stephan Frank, Josef Flammer, Albert Neutzner

**Affiliations:** 1 Department of Biomedicine, University Basel, Basel, Switzerland; 2 Department of Ophthalmology, University Basel, Basel, Switzerland; 3 Division of Neuropathology, Institute of Pathology, University Basel, Basel, Switzerland; Centre for Eye Research Australia, Australia

## Abstract

**Purpose:**

To study the impact of the mitochondrial ubiquitin ligase MARCH5 on mitochondrial morphology and induction of apoptosis using an *in vitro* model of neuronal precursor cells exposed to glaucoma-relevant stress conditions.

**Methods:**

RGC5 cells transfected with expression constructs for MARCH5, MARCH5^H43W^, Dpr1^K38A^ or vector control were exposed to either elevated pressure of 30 mmHg, oxidative stress caused by mitochondrial electron transport chain (ETC) inhibition, or hypoxia-reoxygenation conditions. Mitochondrial morphology of RGC5 cells was analyzed following staining of the mitochondrial marker cytochrome *c* and photoactivatable GFP (PAGFP) diffusion assay. Induction of apoptotic cell death in these cells was determined by analyzing the release of cytochrome *c* from mitochondria into the cytosol and flow cytometry.

**Results:**

Exposure of RGC5 cells to oxidative stress conditions as well as to elevated pressure resulted in the fragmentation of the mitochondrial network in control cells as well as in cells expressing MARCH5. In cells expressing inactive MARCH5^H43W^ or inactive Drp^K38A^, mitochondrial fragmentation was significantly blocked and mitochondrial morphology was comparable to that of control cells under normal conditions. Exposure of RGC5 cells to elevated pressure or oxidative stress conditions induced apoptotic cell death as assessed by cytochrome *c* release and DNA staining, while expression of dominant-negative MARCH5^H43W^ or Drp1^K38A^ did significantly delay cell death.

**Conclusion:**

Preventing mitochondrial fragmentation through interference with the mitochondrial fission machinery protects neuronal cells from programmed cell death following exposure to stressors physiologically relevant to the pathogenesis of glaucoma.

## Introduction

Death of retinal ganglion cells (RGCs) is responsible for vision loss in glaucoma patients. The exact mechanisms causing the demise of RGCs are still under investigation. Different triggers in the various forms of glaucoma probably lead to the observed neurodegenerative process. Elevated intraocular pressure (IOP) is involved in RGC death associated with high-tension glaucoma (HTG) [1], while vascular dysregulation and associated ischemia-reperfusion injury is linked to normal-tension glaucoma (NTG) [2]. Irrespective of the actual trigger and the glaucoma subtype, at its heart, glaucoma is a slowly progressing neurodegenerative disorder. RGC5 cells were used as cellular model. These cells are murine neuronal precursor cells and display certain features such as the expression of specific neuronal marker upon differentiation with various compounds [3].

As mitochondrial dysfunction is generally accepted to be one unifying theme for all neurodegenerative disorders [4], mitochondria and failing mitochondrial function connect the different glaucoma subtypes. Due to the complex architecture of mitochondria and their endosymbiotic origin [5], diverse systems are in place to maintain mitochondrial fidelity [6]. These systems include bacterial type proteases dealing with oxidatively damaged mitochondrial matrix proteins, but also inner mitochondrial membrane-anchored proteases involved in protein processing and protein degradation. Recently, we and others described an important role for the ubiquitin-proteasome system (UPS) and ubiquitin-dependent protein degradation in mitochondrial maintenance [7]. Membrane-anchored ubiquitin ligases such as MULAN/MAPL [8], [9], RNF185 [10] and MITOL/MARCH5 [11], [12], [13] were shown to impact mitochondrial physiology. Furthermore, MARCH5 was demonstrated to promote the degradation of mSOD1 [14], a protein linked to amyotrophic lateral sclerosis, and of polyQ-extended ataxin-3 causative for Machado-Joseph disease [15]. In addition, MARCH5 was connected to the degradation of nitrosylated proteins suggesting a role for this ubiquitin ligase in mitochondrial quality control [16]. Besides the degradation of damaged or superfluous proteins, mitochondrial maintenance critically depends on balanced mitochondrial morphology. Mitochondria form a dynamic network constantly reshaped by the fission and fusion of mitochondrial tubules [17]. MARCH5 was implicated by us and others in the regulation of mitochondrial morphology with inactivation of MARCH5 causing massive mitochondrial elongation due to a block in mitochondrial fission [12]. Mitochondrial fusion is mediated by the mitofusins Mfn1 and Mfn2 that together with Opa1 perform the coordinated fusion of outer and inner mitochondrial membranes. Interestingly, mutations in fusion components are linked to neurodegenerative disorders with Opa1 mutations causative for dominant optic atrophy [18] and mutations in Mfn2 linked to Charcot-Marie-Tooth type 2A disease, a peripheral neuropathy sometimes accompanied by optic degeneration and hearing loss [19]. Division of mitochondria is performed by the dynamin-related protein Drp1 together with hFis1, Mff and MiD49/51 [20], [21], [22]. In a rare case, mutation of Drp1 caused premature death accompanied by microcephaly, persistent lactic acidemia as well as optic degeneration [23], strongly pointing to an underlying mitochondrial etiology. Thus, dynamically balancing and adapting the organelles morphology is an integral part of mitochondrial maintenance and essential for neuronal survival. This is especially true for RGCs, most likely due to their highly specialized anatomy involving non-myelinated parts, their exposure to UV stress, and their – even for neuronal cells – exceptional energy demand [1]. This integration of mitochondrial morphogens into cellular physiology is mirrored in their connection to programmed cell death [24]. Interference with mitochondrial fusion and fission dynamics modulates cell death thresholds with excessive fission sensitizing and blocked fission desensitizing cells to apoptotic stimuli [25], [26]. Consistent with glaucoma being a neurodegenerative disorder and a protective role of mitochondrial fusion in most experimental paradigms, increased expression of the OPA1 fusion protein is protective for RGCs in a mouse model of glaucomatous nerve damage [27].

To investigate the role of the mitochondrial ubiquitin ligase MARCH5 and mitochondrial maintenance during neuronal cell stress, we studied mitochondrial dynamics and induction of cell death in neuronal cells with altered mitochondrial maintenance under physiologically relevant stress conditions.

## Results

**Figure 1:**
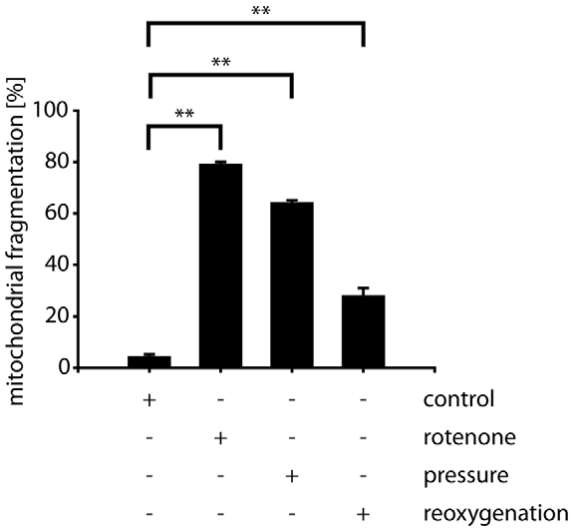
Stress-induced mitochondrial fragmentation in RGC5 cells. Differentiated RGC5 cells were exposed to 0.25 µM rotenone for 12 hours, 30 mmHg elevated pressure for 72 hours or hypoxia-reoxygenation (24 hours 1% oxygen, 2 hours normoxia), fixed and stained using anti-cytochrome *c* antibodies. Mitochondrial morphology was scored visually. Shown are the averages of three independent experiments (>200 cell counted/condition) with error bars representing SEM and * representing p<0.05 and ** representing p<0.01 (Student's t-test).

Exposure of differentiated RGC5 cells to 30 mmHg elevated pressure for three days, to the mitochondrial complex I inhibitor rotenone or to hypoxia-reoxygenation conditions resulted in the fragmentation of the mitochondrial network (Figure 1). While the mitochondrial network in cells kept under ambient pressure displayed normal tubular mitochondrial morphology, in about 60% of cells exposed to elevated pressure mitochondria switched to a fragmented phenotype. For oxidative stress conditions, treatment with rotenone resulted in about 80% of RGCs in mitochondrial fragmentation, while reoxygenation was responsible for fragmented mitochondria in about 30% of cells.

**Figure 2:**
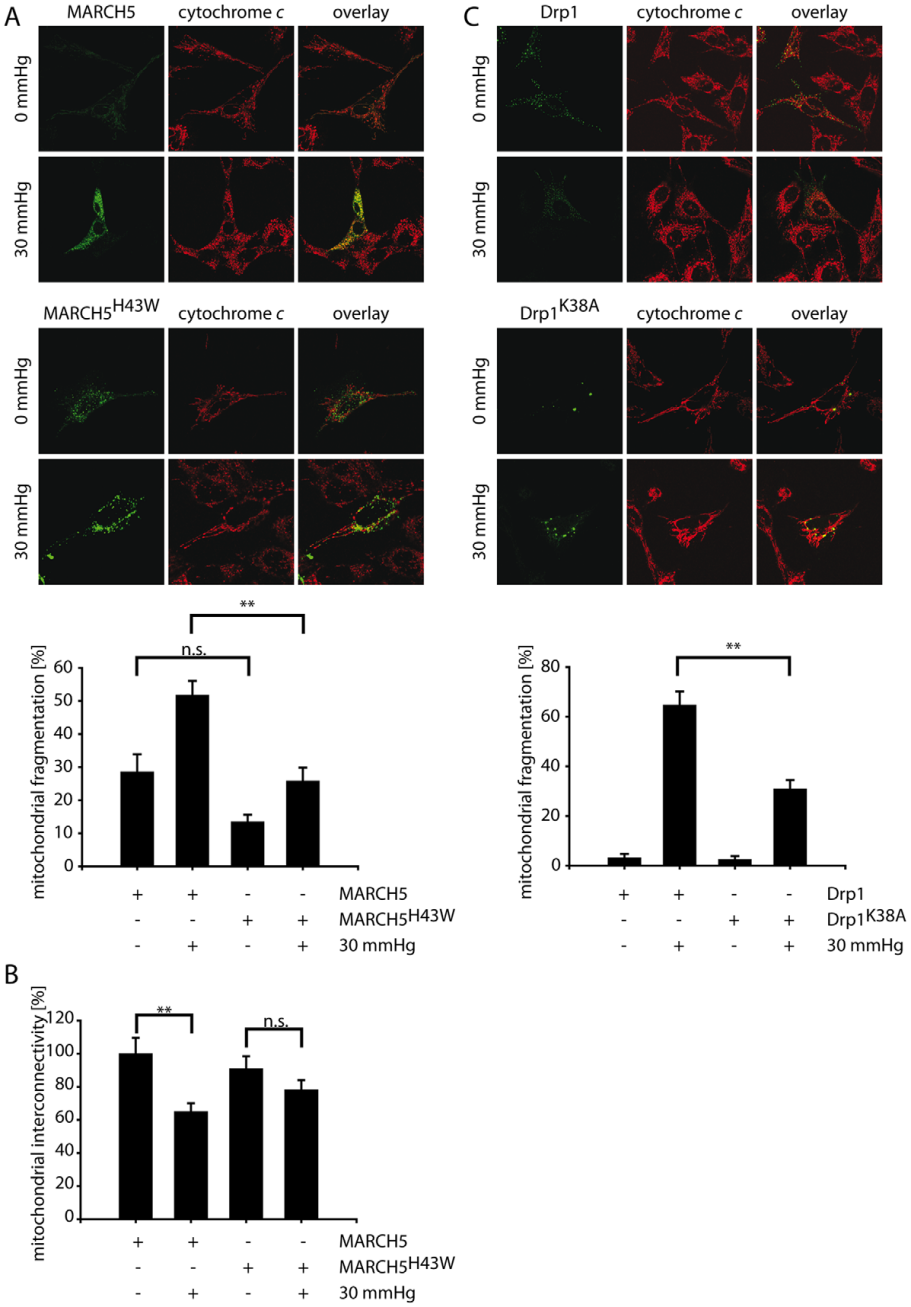
Inactivation of MARCH5 and Drp1 blocks pressure-induced mitochondrial fragmentation. (A) Differentiated RGC5 cells transfected with expression constructs for MARCH5^YFP^ or MARCH5^H43W-YFP^ were exposed for 72 hours to 30 mmHg elevated pressure or left untreated as control. Mitochondrial morphology was assessed following cytochrome *c* staining. The bar graph represents three independent experiments (>200 cell counted/condition) with * marking p<0.05 and ** marking p<0.01 (Student's t-test). Error bars correspond to SEM. (B) RGC5 cells expressing MARCH5 or MARCH5^H43W^ and photoactivatable GFP (PAGFP) were exposed to 30 mmHg for three days or left untreated as control and mitochondrial interconnectivity was measured by PAGFP diffusion after photoactivation and compared to ambient pressure, MARCH5 expressing cells. Analyzed were 20 cells/condition with the error bars representing SEM and ** marking p<0.01 and n.s. marking p>0.05 (Student's t-test). (C) Differentiated RGC5 cells transfected with expression constructs for Drp1^YFP^ or Drp1^K38A–YFP^ were treated as described in A.

As shown in Figure 2, expression of MARCH5 in comparison to control cells did not interfere with pressure-induced mitochondrial fragmentation. Interestingly, expression of a RING-deficient, dominant-negative MARCH5^H43W^ significantly blocked pressure-induced mitochondrial fragmentation and was able to maintain normal mitochondrial morphology in about 75% of cells (Figure 2A). Measuring mitochondrial interconnectivity using a photoactivatable GFP (PAGFP) diffusion assay in cells expressing MARCH5, MARCH5^H43W^ or YFP as control confirmed that expression of MARCH5 did not block pressure-induced changes in mitochondrial interconnectivity, while inactive MARCH5^H43W^ prevented the pressure-induced alteration of the mitochondrial network (Figure 2B). To evaluate the specificity of pressure-induced mitochondrial fragmentation in RGC5 cells, HeLa cells were exposed to identical pressure conditions, and no mitochondrial fragmentation was observed (data not shown). Blocking of mitochondrial fission through expression of dominant-negative Drp1^K38A^ was used to assess the specificity of MARCH5^H43W^ action on mitochondrial morphology following exposure to stress conditions. As shown in Figure 2C, mitochondrial fragmentation was not blocked in RGC5 cells expressing Drp1 following exposure to elevated pressure, while Drp1^K38A^ was capable of blocking organelle fragmentation in about 70% of cells under these conditions.

**Figure 3:**
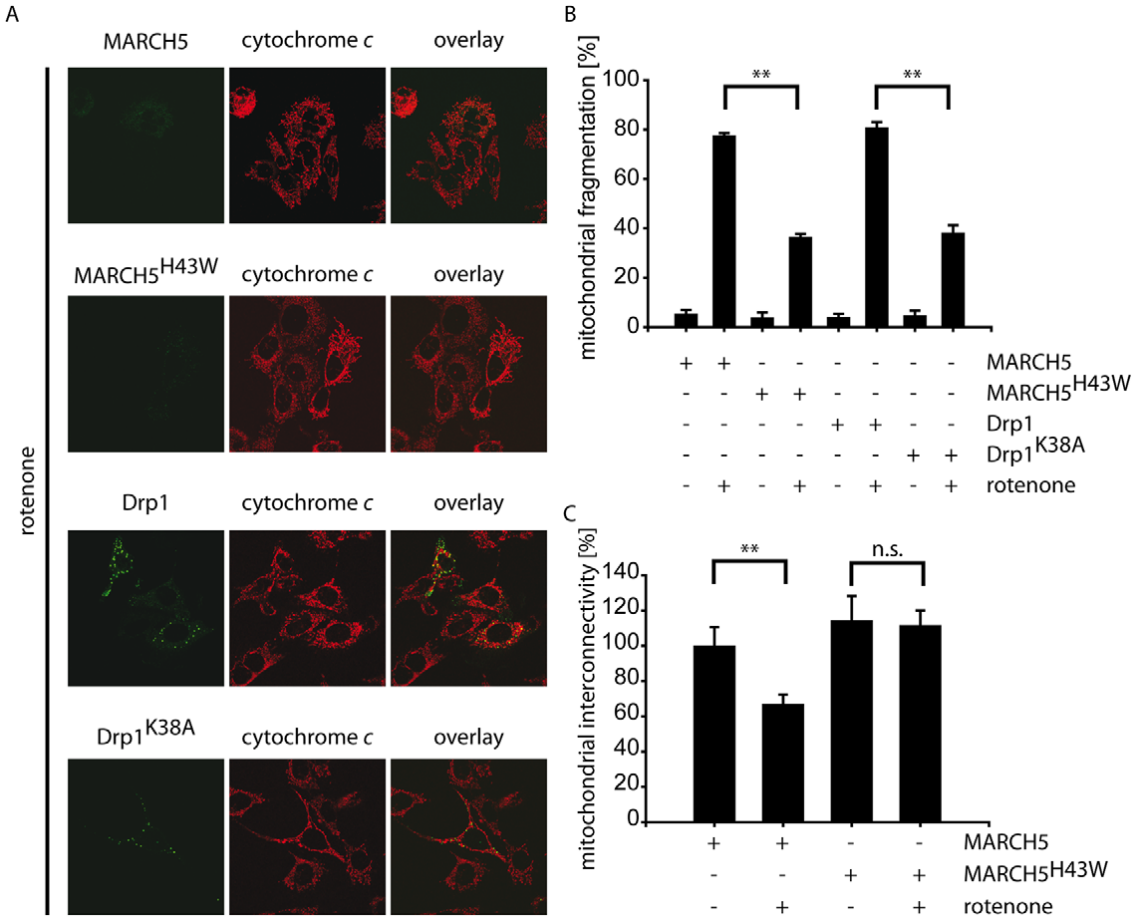
Rotenone-induced mitochondrial fragmentation is reduced following MARCH5 or Drp1 inactivation. (A+B) Differentiated RGC5 cells expressing MARCH5^YFP^, MARCH5^H43W-YFP^, Drp1^YFP^ or Drp1^K38A–YFP^ were exposed to 0.25 µM rotenone for 12 hours prior to fixation and cytochrome *c* staining. Shown is the average of three independent experiments (>200 cell counted/condition), with ** marking p<0.01 (Student's t-test) and error bars representing SEM. (C) RGC5 cells expressing MARCH5 or MARCH5^H43W^ and PAGFP were exposed to 0.5 µM rotenone for 4 hours and mitochondrial interconnectivity was assessed by measuring PAGFP diffusion following photoactivation. Analyzed were 20 cells/condition with error bars representing SEM and ** marking p<0.01 and n.s. marking p>0.05 (Student's t-test).

In an experimental paradigm of oxidative stress, exposure of differentiated RGC5 cells to rotenone, an inhibitor of the complex I of the electron transport chain, did also result in mitochondrial fragmentation (Figure 3A+B) as compared to untreated control cells. Control cells or cells expressing wildtype MARCH5 displayed mitochondrial fragmentation under these conditions. However, ectopic expression of MARCH5^H43W^ interfered with rotenone-induced mitochondrial fragmentation with almost 60% of cells maintaining a tubular mitochondrial network (Figure 3B). Measuring mitochondrial interconnectivity using PAGFP diffusion assay, we confirmed the inhibition of rotenone-induced fragmentation of the mitochondrial network by inactive MARCH5H43w but not wildtype MARCH5 (Figure 3C). Drp1^K38A^ was able to interfere with mitochondrial fragmentation in differentiated RGC5 cells under oxidative stress conditions. While around 80% of control or Drp1-expressing cells displayed mitochondrial fragmentation following rotenone treatment, mitochondrial morphology was tubular in around 60% of Drp1^K38A^-expressing cells (Figure 3A+B).

**Figure 4:**
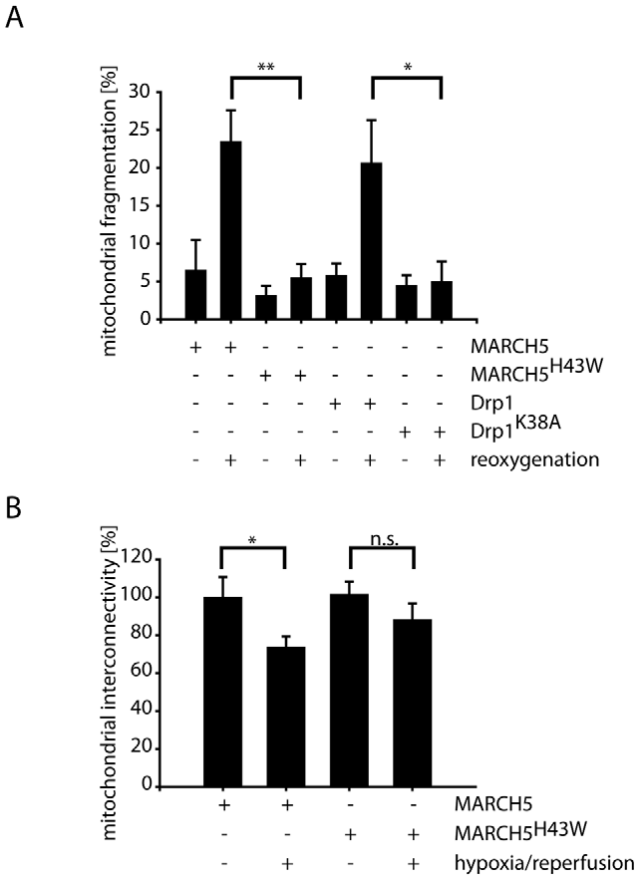
Mitochondrial fragmentation following hypoxia-reoxygenation is ameliorated by inactivation of MARCH5 or Drp1. (A) Differentiated RGC5 cells expressing MARCH5^YFP^, MARCH5^H43W-YFP^, Drp1^YFP^ or Drp1^K38A–YFP^ were cultured in the presence of low oxygen (1%) for 24 hours followed by normoxia for 2 hours. Mitochondrial fragmentation was analyzed following cytochrome *c* staining in three independent experiments (>200 cell counted/condition). Error bars represent SEM, p-Values for Student's t-test are marked with * (p<0.05) or ** (p<0.01). (B) RGC5 cells expressing MARCH5 or MARCH5^H43W^ and PAGFP were stressed using hypoxia-reoxygenation and mitochondrial interconnectivity was assessed by measuring PAGFP diffusion following photoactivation. Analyzed were 20 cells/condition with error bars representing SEM and ** marking p<0.01 and n.s. marking p>0.05 (Student's t-test).

Re-oxygenation following exposure to low oxygen atmosphere mimicking ischemia-reperfusion conditions induces mitochondrial fragmentation in differentiated RGC5 cells (Figure 1 and 4). Re-oxygenation-induced mitochondrial fragmentation was not blocked in cells ectopically expressing MARCH5 or Drp1 when compared to transfected control cells (Figure 4). Interestingly, ectopic expression of MARCH5^H43W^ or Drp1^K38A^ did completely block mitochondrial fragmentation under these conditions (Figure 4A). We confirmed this observation in MARCH5 or MARCH5H43W expressing cells treated with hypoxia-reperfusion using PAGFP diffusion assay (Figure 4B).

**Figure 5:**
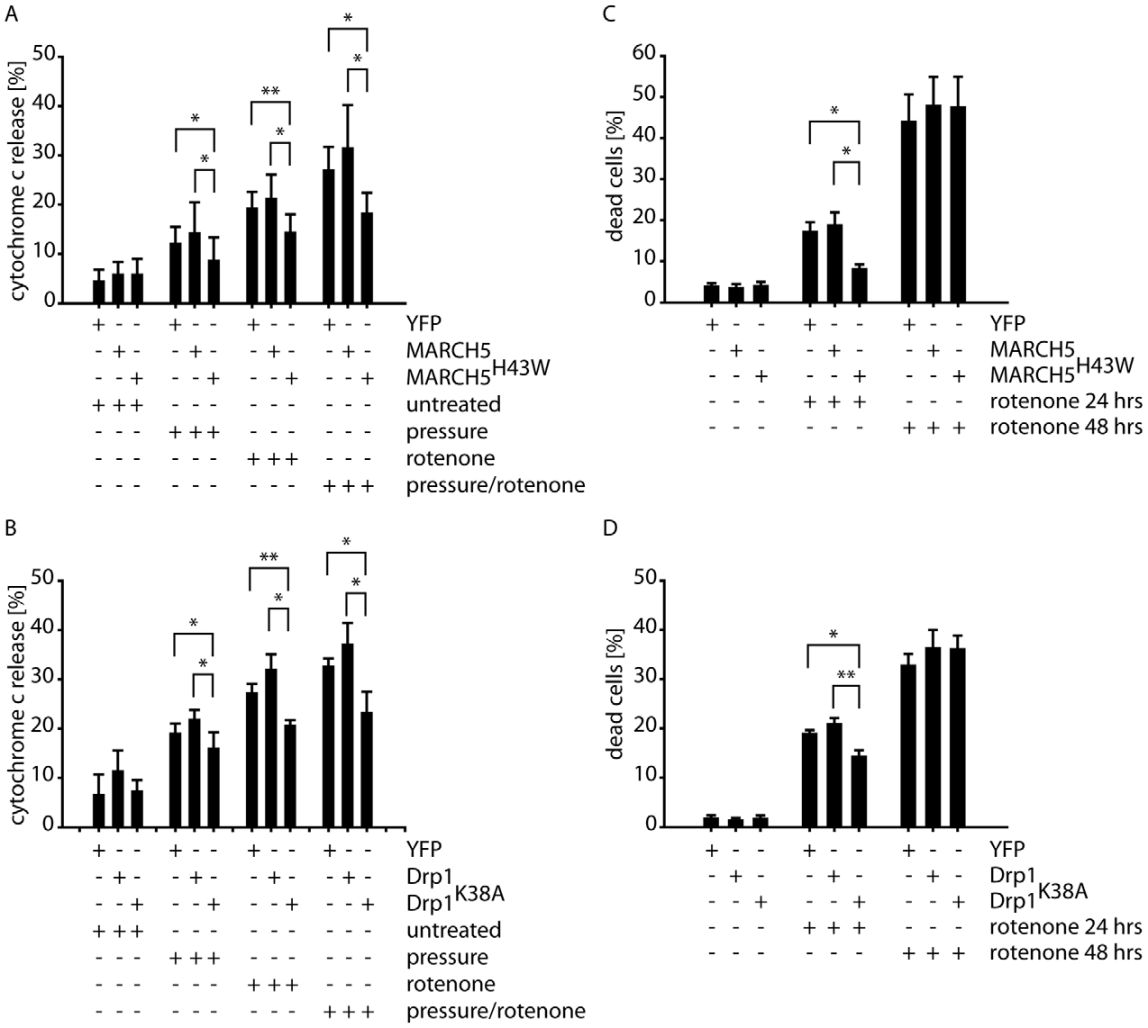
Inactivation of MARCH5 or Drp1 delays induction of apoptosis and cell death. RGC5 cells expressing (A) MARCH5^YFP^, MARCH5^H43W-YFP^ or YFP as control or (B) Drp1^YFP^ or Drp1^K38A–YFP^ or YFP as control were exposed to 100 mmHg for 24 hours, 1 µM rotenone for 6 hours or combined 100 mmHg pressure and 1 µM rotenone in the presence of the pan-caspase inhibitor zVAD-fmk. Following treatment, cells were fixed and cytochrome *c* release from mitochondria into the cytosol was assessed by fluorescence microscopy (>200 cell counted/condition). The bar graphs represent four independent experiments with * marking p<0.05 and ** marking p<0.01 (Student's t-test). RGC5 cells expressing (C) MARCH5^YFP^, MARCH5^H43W-YFP^ or YFP as control or (D) Drp1^YFP^ or Drp1^K38A–YFP^ or YFP as control were exposed to 1 µM rotenone for 0, 24 or 48 hours and the amount of dead cells was measured by flow cytometry following 7-AAD staining of DNA.

Furthermore, we assessed whether modulation of mitochondrial morphology through expression of Drp1^K38A^ or MARCH5^H43W^ altered the sensitivity of differentiated RGC5 cells to apoptotic stimuli. To this end, RGC5 cells were exposed either to 100 mmHg elevated pressure for one day, rotenone alone, or a combination of elevated pressure and rotenone, and apoptotic induction in the presence of pan-caspase inhibitor was assessed by counting the release of cytochrome *c* from mitochondria. As shown in Figure 5, exposing RGC5 to these stress conditions leads to the induction of apoptotic cell death in control cells and in cells expressing wildtype MARCH5 (Figure 5A) or Drp1 (Figure 5B). Interestingly, expression of MARCH5^H43W^ (Figure 5A) or Dpr1^K38A^ (Figure 5B) resulted in significant suppression of apoptotic cell death induction.

To further examine the extent to which inactivation of mitochondrial fission by mutant MARCH5 or mutant Drp1 delays apoptosis, we measured cell death following prolonged exposure to oxidative stress conditions. To this end, cells expressing MARCH5 or MARCH5^H43W^ (Figure 5C) or Drp1 or Drp1^K38A^ (Figure 5D) or YFP as control were treated for 24 or 48 hours with 1 µM rotenone or left untreated. The amount of accumulating dead cells following this treatment was measured using flow cytometric analysis of 7-aminoactinomycin D (7-AAD) exclusion. Interestingly, expression of wildtype MARCH5 (Figure 5C) or wildtype Drp1 did not interfere with the progression of cell death, while MARCH5^H43W^ or Drp1^K38A^ did significantly diminish cell death during 24 hours of rotenone treatment in comparison to control or MARCH5 or Drp1 expressing cells, respectively. However, at 48 hours of rotenone treatment, neither expression of MARCH5^H43W^ nor Drp1^K38A^ did significantly alter the accumulation of 7-AAD-positive cells.

## Discussion

Mitochondrial fidelity is important for neuronal survival, with failing mitochondria and mitochondria-mediated cell death involved in neuronal degeneration. Therefore, maintaining mitochondria in a healthy and functional state is essential for neuronal survival. Mitochondrial surveillance and repair is performed by a multi-tiered system involving specialized mitochondrial proteases, the ubiquitin-proteasome system, properly balanced mitochondrial dynamics, but also cell death mechanisms [4]. The mitochondrial ubiquitin ligase MARCH5 is involved in mitochondrial maintenance through the clearing of mutated, damaged mitochondrial proteins but also through regulating mitochondrial fission [12], [14], [15], [16]. Both of these functions of MARCH5 are important for neuronal survival. Degradation of proteins such as mSOD1 or polyQ-ataxin-3 associated with amyotrophic lateral sclerosis or Machado-Joseph disease [15], respectively, by MARCH5 was shown to exert neuroprotective functions. Furthermore, maintenance of a plastic mitochondrial network through balanced mitochondrial fission and fusion was shown to be important for neuronal survival. For example, mfn2^−/−^ mice display severe loss of Purkinje cells likely caused by unbalanced mitochondrial dynamics [28]. Furthermore, in humans, impaired mitochondrial fusion caused by mutations in Opa1 and Mfn2 are associated with dominant optic atrophy or Charcot-Marie-Tooth disease 2A (CMT2A), respectively. We now identified a role for MARCH5 in the death of retinal ganglion cells caused by stress conditions that are relevant for glaucoma progression. We found that modulation of mitochondrial morphology through MARCH5 depends on its regulation of Drp1 recruitment to the OMM. For mitochondrial fission to occur cytosolic Drp1 has to assemble into fission complexes on mitochondria in a MARCH5-dependent manner; expression of dominant-negative MARCH5^H43W^ locks Drp1 in fission-incompetent mitochondrial division complexes. The observed block in mitochondrial fission in differentiated MARCH5^H43W^-expressing RGC5 cells following exposure to elevated pressure, oxidative stress or to ischemia-reperfusion conditions is consistent with the known role of MARCH5 in the regulation of mitochondrial morphology [12]. This notion is supported by our findings that expression of dominant-negative Drp1^K38A^ but not of wildtype Drp1 blocks mitochondrial fragmentation following elevated pressure, oxidative stress or re-oxygenation in a comparable manner. This finding is consistent with MARCH5 acting upstream of Drp1 in the regulation of mitochondrial fission. Interestingly, inactivation of MARCH5 blocks mitochondrial fragmentation under the tested stress conditions as effectively as dominant-negative inaction of Drp1 function. While the exact role of MARCH5 during mitochondrial fission is still unclear, our data suggest a strong dependence of Drp1-mediated fission on MARCH5 activity.

Whereas MARCH5 is neuroprotective under conditions of insufficient protein quality control as seen e.g. in Machado-Joseph disease [15], its expression has no beneficial effect under glaucoma-relevant stress conditions. In contrast, inactivation of MARCH5 function through dominant-negative action of MARCH5^H43W^ exerts anti-apoptotic activity, slows down cell death and exerts a neuroprotective function. Thus, under the conditions tested here, the function of MARCH5 in mitochondrial division is predominant. In addition, elevated pressure, oxidative stress and ischemia-reperfusion do not seem to cause damage to mitochondrial proteins that are under the surveillance of the mitochondrial ubiquitin ligase MARCH5. One might speculate that inhibition of mitochondrial fragmentation through pharmacological inactivation of MARCH5 might be beneficial under certain circumstances, similar to what has been shown for Drp1, where pharmacological inhibition of mitochondrial fission by the small molecule Drp1 inhibitor mdivi-1 proved neuroprotective [29].

## Conclusions

Taken together, the versatile mitochondrial ubiquitin ligase MARCH5 impacts neuronal survival in various ways either through the degradation of damaged mitochondrial proteins or by modulating mitochondrial morphology. In case of glaucoma-related stressors and retinal ganglion cells, the role of MARCH5 as fission regulator outweighs its other roles in maintaining mitochondrial proteostasis.

## Methods

### Cell culture

Immortalized neuronal precursor cells (RGC5) were cultured in high glucose DMEM, supplemented with 10% fetal bovine serum, 2 mM L-glutamine, MEM non-essential amino acid (Sigma-Aldrich) and incubated in a humidified incubator at 5% CO_2_ and 37°C. RGC5 cells were received as gift from Neville Osborne (University of Oxford) and tested as of mouse origin by PCR [30] and are thus considered neuronal precursor cells instead of retinal ganglion cells as originally proposed [31]. Where stated, cells were cultured in media containing 1% FBS. For immunocytochemistry, cells were seeded onto sterilized 18 mm diameter glass coverslips in 6-well plates at a density of 1×10^5^ cells/well. Cells were transfected using Effectene (Qiagen) at a ratio of 1∶10 (DNA:transfection reagent) following manufacturer's instructions.

To induce differentiation of RGC5 cells, cells were seeded into appropriate vessels and allowed to attach for 6 h in media containing 10% FBS before changing to serum-free media for 24 hours. Afterwards, cells were incubated in FBS medium containing *s*uccinyl-concanavalin A (50 µg/ml) for 3 days [31].

To expose RGC5 cells to elevated pressure, cells were placed into a custom-made pressure chamber [32] and exposed to 30 mmHg (72 h) or 100 mmHg (24 h) above ambient pressure. To induce oxidative stress, RGC5 cells were treated with the mitochondrial complex I inhibitor rotenone (Sigma-Aldrich) at the indicated concentrations. To expose RGC5 cells to hypoxia-reoxygenation, cells were cultured at 1% oxygen/5% CO_2_ for 24 hours following exposure to normoxia for 2 hours prior to fixation.

To prevent progression of apoptosis following stress treatment, cells were pre-treated with 50 µM of the pan-caspase inhibitor zVAD-fmk (PeptaNova).

### Immunocytochemistry

To assess mitochondrial morphology and release of cytochrome *c*, RGC5 cells were fixed using methanol-free electron microscopy grade 4% paraformaldehyde in PBS (Pierce) for 15 minutes at RT, permeabilized for 15 minutes at RT using 0.15% Trixon X-100 in PBS and blocked for 1 h in 10% BSA in PBS. To stain for cytochrome *c*, cells were incubated with mouse anti-cytochrome *c* antibody (1∶1000, Abcam 556432) and Alexa546-conjugated anti-mouse antibodies (1∶500, Invitrogen). Samples were mounted using mounting medium (Vectashield, H-1000) and observed using immunofluorescence microscopy (Olympus BX 61, 60×/1.35 objective) or confocal microscopy (Zeiss Meta710, 63×/1.4 objective).

### Analysis of mitochondrial morphology

Mitochondrial morphology was judged visually following cytochrome *c* staining relying on observer experience. Mitochondria were judged “normal” if appearance resembled control cells with mostly middle sized mitochondria and only some smaller fragments. Mitochondria were scored as fragmented if the majority of mitochondria (>90%) were either round or slightly elongated (length <2x width). Mitochondria were scored as elongated if only a minor fraction of mitochondria were of “normal” size and organelle continuously extended across the entire length/width of the cell. Each experiment was done independently at least three times and a minimum of 200 cells/condition were counted by an unblinded observer.

To more quantitatively assess mitochondrial morphology, RGC-5 cells were cultured on chambered cover glass and co-transfected in a 1∶3 ratio with mitochondria-targeted photoactivatable GFP (mito-PAGFP) and MARCH5 or MARCH5^H43W^ expression constructs. The PAGFP assay was performed on a LSM710 confocal microscope (Zeiss) equipped with a 63x objective using ZEN software. For photoactivation, a 75 pixel wide circle was randomly selected. Activation was performed using the 405 nm laser line (100% output) at zoom 4, 100 μs pixel dwell time and 2 iterations. One z-stack, both before and after activation, was acquired (5 images at a 0.67 μm interval). LSM Image Browser (Zeiss, v. 4.2.0.121) and ImageJ (NIH, v. 1.45 s) were used to analyze mitochondrial interconnectivity as follows: the activated area (75 pixel) was masked and a maximum intensity projection of the z-stack was created using LSM Image Browser. ImageJ was used to measure the total area of fluorescent mitochondria outside the photoactivated area (steps performed in ImageJ: threshold adjustment so that only photoactivated mitochondria were visible, then median filter radius 2 followed by analyze particles size 25-infinity). Twenty randomly selected cells were analyzed for each condition. The measured area was compared between different treatment groups relative to untreated MARCH5-expressing cells.

### Flow cytometric analysis of cell death

RGC5 cells expressing MARCH5^YFP^, MARCH5^H43W–YFP^ or YFP as control were treated with 1 µM rotenone for 24 or 48 hours or left untreated. Attached and floating cells were harvested, stained with 1 µg/ml 7-aminoactinomycin D and analyzed by flow cytometry (CyAn ADP, Beckman Coulter). 7-AAD fluorescence (FL4) was measured in YFP positive cells to determine percentage of dead cells.

### Statistical Analysis

All experiments were performed at least three times. For each experiment a minimum of 200 cells/condition were counted. Statistical analysis was done using unpaired, two-tailed Student's t-test as implemented in Microsoft Excel. A p-Value of <0.05 or smaller was considered statistically significant and is marked with *, while p-Values of <0.01 are marked with **. Error bars represent the standard error of the mean (SEM). For PAGFP diffusion assay, 20 cells per condition were measured and statistical analysis was performed as described above.
